# MLKL Mediated Necroptosis Accelerates JEV-Induced Neuroinflammation in Mice

**DOI:** 10.3389/fmicb.2017.00303

**Published:** 2017-02-28

**Authors:** Peiyu Bian, Xuyang Zheng, Li Wei, Chuantao Ye, Hong Fan, Yanhui Cai, Ying Zhang, Fanglin Zhang, Zhansheng Jia, Yingfeng Lei

**Affiliations:** ^1^Department of Infectious Diseases, Tangdu Hospital, Fourth Military Medical UniversityXi'an, China; ^2^Department of Obstetrics and Gynecology, Xijing Hospital, Fourth Military Medical UniversityXi'an, China; ^3^Department of Neurobiology and Collaborative Innovation Center for Brain Science, School of Basic Medicine, Fourth Military Medical UniversityXi'an, China; ^4^Department of Anesthesiology, Xijing Hospital, Fourth Military Medical UniversityXi'an, China; ^5^Department of Microbiology, School of Preclinical Medicine, Fourth Military Medical UniversityXi'an, China

**Keywords:** Japanese encephalitis virus, neuronal death, necroptosis, MLKL, inflammation

## Abstract

Japanese encephalitis virus (JEV) is the most prevalent cause of viral encephalitis in Asia and the western Pacific. Neuronal death caused by JEV infection and inflammation induced cytotoxicity leads to progression and deterioration of Japanese encephalitis (JE). Mixed-lineage kinase domain-like protein (MLKL) mediated necroptosis is a newly discovered pathway of programmed cell death and participates in many inflammatory diseases. In this study, we demonstrated for the first time that necroptosis was involved in the neuronal loss during JE via immune-electron microscopy and immunochemistry. The expression of MLKL in neurons was upregulated in presence of JEV infection *in vitro* and *in vivo*. Deletion of MLKL alleviated the progression of JE and decreased the level of inflammatory cytokines in mice model. Taken together, this study provides evidence for the participation of necroptosis in the pathogenesis of JEV infection.

## Introduction

Japanese encephalitis virus (JEV) is the most prevalent cause of viral encephalitis worldwide. More than 67,900 cases of JE are reported annually, among which approximately 30% are fatal and 50% suffer from permanent neuropsychiatric sequelae (Wang and Liang, 2015). JEV infection causes devastating and fatal neuroinflammation characterized by neuronal destruction accompanied with intense microgliosis, strogliosis, and production of various inflammatory cytokines (Biswas et al., 2010; Chen et al., 2010). Neurons can be destroyed directly by JEV infection and indirectly by inflammation mediated cytotoxicity (Ghoshal et al., 2007; Myint et al., 2014). Then the massive neuronal death contributes to the progression and deterioration of Japanese encephalitis (JE) (Myint et al., 2014).

Necroptosis is a newly discovered pathway of programmed cell death (Galluzzi and Kroemer, 2008). Unlike the uncontrolled accidental cell death, necroptosis is regulated by classical necrosome comprising receptor-interacting protein kinase 1 (RIPK1), RIPK3, mixed-lineage kinase domain-like protein (MLKL) through TNF/TNFR1 signaling or other stimuli (Vandenabeele et al., 2010; Sun et al., 2012; Vanden et al., 2014). In addition, DNA-dependent activator of IFN regulatory factors (DAI) is also identified to induce the RIPK1-independent RIPK3-dependent necroptosis by recognizing viral double-stranded DNA and recruiting RIPK3 directly through the RHIM domain (Upton et al., 2012; Sridharan and Upton, 2014; Kuriakose et al., 2016). MLKL is the executor of necroptosis after being recruited and phosphorylated by RIPK3. MLKL recruits Ca^2+^ and Na^+^ ion channels and form pores at the plasma membrane, leading to the rupture of cells. (Cai et al., 2014; Dondelinger et al., 2014; Wang et al., 2014). Massive damage-associated molecular patterns (DAMPs) released from the disintegrated cells trigger inflammatory response (Kaczmarek et al., 2013). Overproduction of inflammatory cytokines (e.g., TNF-α) from activated microglia exposed to DAMPs may induce more cell death which forms the vicious cycle of necroptosis-induced inflammation (Pasparakis and Vandenabeele, 2015).

Previous studies have identified that apoptosis and autophagy are involved in the neuronal loss of JE, which are reported to be effective intervention targets to alleviate the progression of JE (Sharma et al., 2014; Lyoo et al., 2015; Huang et al., 2016). Besides, massive neuronal necrosis is also observed in the human fatal cases and animal models of JEV infection. During JE, TNF-α is the main culprit in the neurotoxic cascade and provokes glial activation as well as subsequent neuroinflammation (Chen et al., 2012). Interestingly, TNF-α is just the classical initiator of necroptosis through TNF-α/TNFR1/RIPK1/RIPK3/MLKL signaling in many central nervous system (CNS) diseases (Cai et al., 2014; Wang et al., 2014). Necroptosis was also reported to be activated during viral infection such as herpes simplex virus (HSV), influenza A virus (IAV) and murine cytomegalovirus (MCMV) by death cytokines such as TNF or virus itself (Upton et al., 2012; Huang et al., 2015; Kuriakose et al., 2016). However, it is still unknown whether necroptosis was involved in the development of JE. In this study, we found that MLKL mediated necroptosis was activated in JE and MLKL deleted mice showed ameliorated progression of JE compared with wild mice. Taken together, this study provides direct evidence for the roles of necroptosis in the pathogenesis of JEV infection.

## Materials and methods

### Ethics statement

All animal experiments were approved by the Animal Care and Use Committee of the Fourth Military Medical University.

### MLKL^−/−^ mice

The MLKL^+/−^ *C57BL/6* mice were kind gifts from Professor Jiahuai Han and Dr. Jianfeng Wu (State Key Laboratory of Cellular Stress Biology and School of Life Sciences, Xiamen University) and were kept in a specific pathogen-free (SPF) facility. New-born mice toe DNA samples were amplified through PCR with PrimeStar (Takara, Japan) and the products were analyzed by agarose gel electrophoresis for the screening of WT, MLKL^+/−^, MLKL^−/−^ descendants (Wu et al., 2013). To build the JEV infected models, Wild and MLKL^−/−^ mice (4–6 W) were administered with JEV 5 × 10^7^ PFU/20 g in 200 μl phosphate buffered saline (PBS) intraperitoneally. For the control, 200 μl PBS was injected intraperitoneally.

### Cells and virus

JEV SA-14 strain was propagated in the mosquito cell line *C6/36*. The supernatant containing JEV was concentrated as follows, the supernatant of JEV-infected C6/36 cell was collected and centrifuged at 8,000 g for 30 min at 4°C to remove the cell debris. Next, the supernatant was ultra-centrifuged at 40,000 g for 4 h at 4°C and the viral pellet was resuspended with PBS at 100:1 (supernatant: suspension) after removing the supernatant thoroughly. After being filtered through 0.22 μm filters, the virus suspension was titrated by conventional plaque assay and stored at −80°C (Lee et al., 2014).

The neuroblast cell line Neuro2a was bought from ATCC and kept in DMEM (Gibco, Grand Island, NY, USA) containing 10% fetal bovine serum (FBS, Gibco, Grand Island, NY, USA).

### Immuno-electron microscopy

The immune-electron microscopy was performed as described (Fan et al., 2016). Briefly, at 5 days post infection (dpi), mice were anesthetized with 2% pentobarbital sodium (0.1 ml/10 g body weight) and perfused with 0.01 M PBS followed by 4% paraformaldehyde (PFA) containing 0.05% glutaraldehyde for 30 min. The brains were removed and fixed in the 4% PFA for 3 h. Tissue sections of 50 μm were prepared with a vibratome and cryoprotected by 30% sucrose. After one freeze-thaw treatment, the sections were blocked with 5% bovine serum albumin (BSA) and 5% normal goat serum for 4 h, then incubated with anti-MLKL antibody (Merck Millipore, Billerica, MA, USA) for 24 h, after rinsed thoroughly and then incubated with goat anti-rat IgG conjugated to 1.4 nm gold particles (1:100, Nanoprobes) at room temperature (RT) overnight. After rinsing three times, the sections were fixed with 2% glutaraldehyde for 45 min. Silver enhancement was performed in the dark with an HQ Silver Kit (Nanoprobes) for 15 min. The sections were further fixed with 0.5% osmium tetroxide, dehydrated with graded ethanol, replaced with propylene oxide, and flat-embedded in Epon 812. The MLKL-immunoreactive areas were selected, trimmed under a stereomicroscope and mounted onto blank resin stubs for ultrathin sectioning. Ultrathin sections (70–80 nm) were prepared on an LKB Nova Ultratome (Bromma). After being counterstained with uranyl acetate and lead citrate, the sections were examined under a JEM-1230 electron microscope (JEM, Japan).

### Propidium iodide (PI) staining *in vivo*

PI (4 mg/ml, Sigma, St. Louis, MO, USA) was prepared in 0.9% NaCl. At 5 dpi, mice were administered with PI intraperitoneally (100 μl/20 g weight). One hour later, mice were anesthetized with 2% pentobarbital sodium and then perfused with 0.01 M PBS (50 ml) followed by 4% PFA for 30 min. Protected from light, the brains were removed and fixed in the 4% PFA for 3 h subsequently cryoprotected by 30% sucrose for 48 h. Brain sections of 10 μm were prepared with a vibratome and the nuclei were counterstained by DAPI (100 ng/ml, Sigma, St. Louis, MO, USA).

### Immunohistochemical staining (IHC)

At 5 dpi, mice were anesthetized with 2% pentobarbital sodium and then perfused with 0.01 M PBS followed by 4% PFA for 30 min. The brains were removed and fixed in the 4% PFA for 3 h subsequently cryoprotected by 30% sucrose for 48 h. Tissue sections of 10 μm were prepared with a vibratome. After being fixed with 4% PFA and blocked with 3% BSA containing 0.1% Triton X-100, the slides were incubated with primary antibodies anti-NeuN (abcam, Cambridge, MA, USA), anti-JEV (prepared in our lab), and anti-MLKL (Millipore, Billerica, MA, USA) diluted with PBS containing 0.1% Triton X-100 and 1% BSA for 16 h. After washing, the sections were incubated with the corresponding secondary antibodies (see Supplementary Table 1) for 1 h at RT. The nuclei were counterstained with DAPI, and coverslips were placed on the samples with 50% glycerol in PBS.

### Neuro2a cells infection

Neuro2a cells were plated in 24-well plates containing glass slides at the density of 6 × 10^4^ /well overnight. Then cells were infected with JEV (MOI = 1). After adsorption for 1 h, the virus suspension was removed, and fresh DMEM was added. After 24 h, the slides were fixed with 4% PFA for 30 min and blocked with 3% BSA for 1 h. Then IHC was conducted for MLKL and JEV as above.

Neuro2a cells were plated in 6-well plates at the density of 2 × 10^5^/well in DMEM containing 10% FBS overnight. Then cells were infected with JEV as above at different MOI (0.1, 1, and 5) and harvested at different time points (12, 24, 48 h after infection) for qRT-PCR and western blotting.

### qRT- PCR

WT and MLKL^−/−^ mice were euthanatized and perfused with PBS at 5 dpi, then whole brain of each mouse was harvested and stored at −80°C. Total RNA from mouse brains and Neuro2a cells was extracted with RNAfast1000 (PIONEER, China). The cDNA was prepared by reverse transcription with the total RNA as template using the PrimeScript RT reagent Kit (TaKaRa, Japan), qRT-PCR experiments were carried out using SYBR Green Real-Time PCR Master Mix (TaKaRa, Japan) according to the manufacturer's instructions. Each quantitative PCR reaction was performed in triplicate and the mean threshold cycle (Ct) value for each sample was used for data analysis. The level of mRNA expression was normalized with β-actin. The primers used in this study are as follows:

JEV (forward, 5′- AGACAAGCAGATCAACCACCATT-3′ and reverse, 5′- CCCTCCAATAGAGCCAAAGTCC -3′).
TNF-α (forward, 5′- CTGAACTTCGGGGTGATCGGT -3′ and reverse, 5′-ACGTGGGCTACAGGCTTGTCA-3′).
IFN-γ (forward, 5′- GGCCATCAGCAACATAAGCGT -3′ and reverse, 5′-TGGGTTGTTGACCTCAAACTTGGC-3′).
CCL-2 (forward, 5′- CAAGAAGGAATGGGTCCAGA -3′ and reverse, 5′-GCTGAAGACCTTAGGGCAGA-3′).
IL-1β (forward, 5′- TCCAAGAAAGGACGAACATTCG -3′ and reverse, 5′-TGAGGACATCTCCCACGTCAA-3′).
MLKL (forward, 5′- GGATTGCCCTGAGTTGTTGC -3′ and reverse, 5′-AACCGCAGACAGTCTCTCCA-3′).
β-actin (forward, 5′- TGACGGGGTCACCCACACTG -3′ and reverse, 5′-AAGCTGTAGCCGCGCTCGGT-3′).

### Western blotting

Total protein from the brain of each mouse or Neuro2a cells was extracted with RIPA buffer and quantified using a Protein Reagent Assay BCA Kit (Thermo, Waltham, MA, USA). Thirty micrograms of protein from each sample was loaded and electrophoresed using 10% SDS-PAGE gels and then transferred onto a polyvinylidene difluoride (PVDF) membrane (Millipore, Billerica, MA, USA). After being blocked with 3% BSA at RT for 60 min, the membranes were incubated with primary antibodies anti-MLKL (Millipore, Billerica, MA, USA), pMLKL (abcam, Cambridge, MA, USA) and β-actin (Proteintech, China) overnight at 4°C. Then, the blots were incubated with secondary antibody (see Supplementary Table 1) for 2 h at RT. The blots were visualized using an Infrared Imaging System (Odyssey, LI-COR, NE Lincoln, USA).

### ELISA assay

Serum was collected from each mouse of each group at 5 dpi. The level of IL-1β, CCL-2, IFN-γ, and TNF-α was tested with Mouse ELISA Kit (AMEKO, China) and the absorbance was measured at 450 nm. The concentration was determined according to the standard curve.

### Statistical analysis

All statistical analyses used GraphPad Prism version 6.01 software. Statistical differences were determined using the Student's *t*-test. *P*-values < 0.05 were considered significant (^*^P < 0.05, ^**^P < 0.01, and ^***^P < 0.001).

## Results

### Nerve cells undergo necrosis during JE in mice model

**Figure 1:**
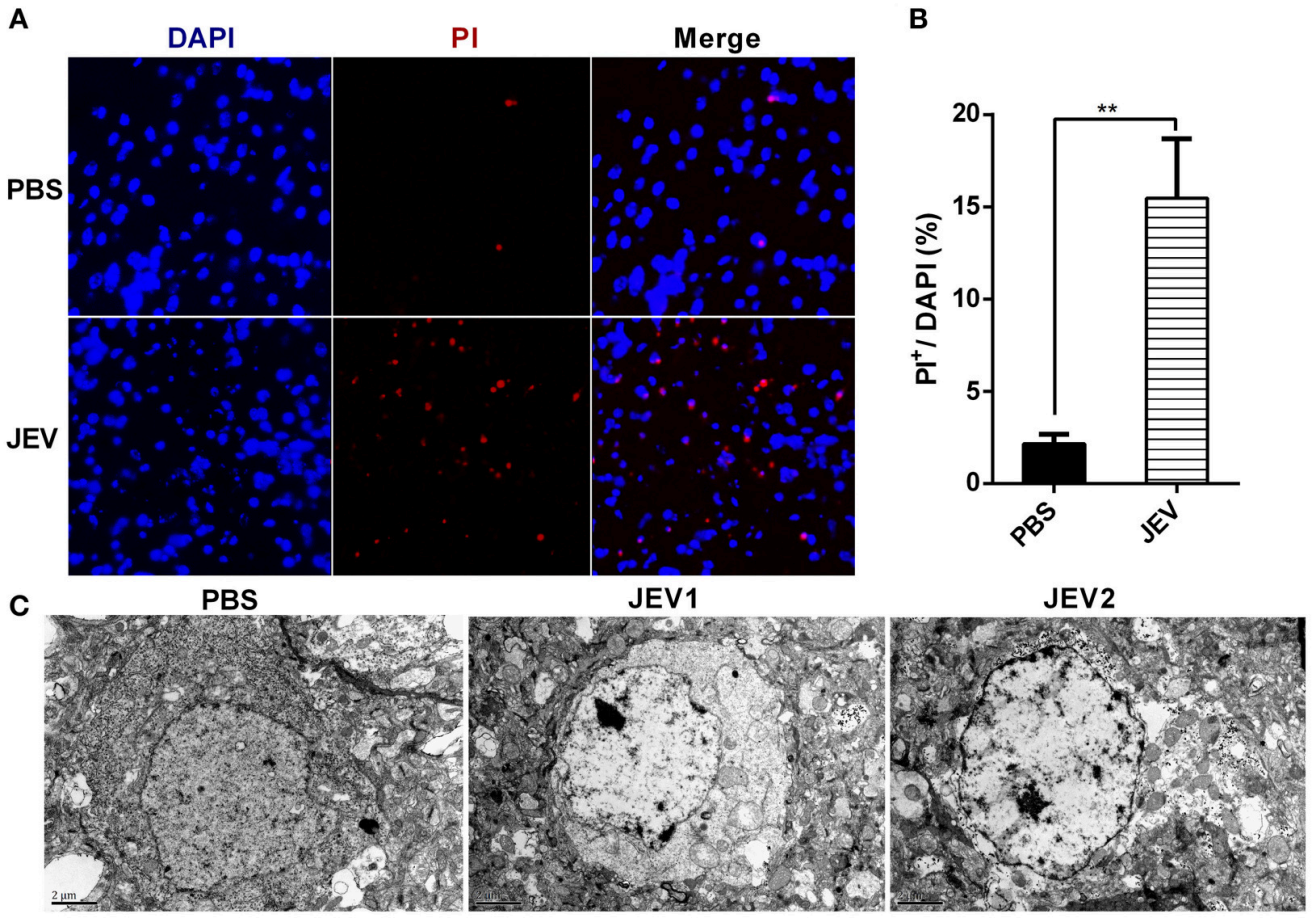
**Nerve cells undergo necrosis during JE in mice model**. C57BL/6 mice were infected i.p. with PBS or JEV 5 × 10^7^ PFU in 200 μl PBS/20 g per mouse. From 5th dpi, JEV infected mice developed body weight loss and early clinical signs such as piloerection, and physical limitation. Brains from each mouse were harvested for further experiment at 5 dpi. **(A)** The representative images of PI staining of brain sections from PBS or JEV administrated mice (200x). **(B)** The intensity of PI positively stained cells of each group were analyzed with Image J. (Data represents mean ± SEM. PBS = 2, JEV = 3; 3 sections per mouse, 5 fields per section, ^**^P < 0.01). **(C)** Micrographs of normal and JEV infected mouse brain cells. In PBS group, the morphology of brain cells. In JEV infected mice, most of brain cells showed classical necrotic morphology with clumps of chromatin, swollen mitochondria and plasma membrane disintegration (PBS = 1, JEV = 2).

It is known that JEV infection causes neuronal cell death directly by JEV infection and indirectly by inflammation mediated cytotoxicity. Besides apoptosis, neuronal necrosis is also found in autopsy tissues from human fatal cases (German et al., 2006). Compared with the orderly disassembly of apoptotic cells, necrotic cells are disintegrated accompanied with massive release of DAMPs, which can trigger more serious inflammation (Kaczmarek et al., 2013). In this study, necrosis was detected in mice infected with JEV at 5 dpi through PI staining and electron microscopy. At 5 dpi, massive antigen of JEV was detected in JEV infected mouse brains (see Supplementary Figure 1). There were a large number of PI positive cells in the brain sections of JEV infected mice compared with PBS treated mice (Figures 1A,B). In the microstructure of brain cells, there were many cells showing classical necrotic morphology with clumps of chromatin, swollen mitochondria and plasma membrane disintegration in JEV infected group (Figure 1C). As a result, nerve cells underwent massive necrotic cell death in the murine model of JE.

### MLKL mediated necroptosis is involved in JE

**Figure 2:**
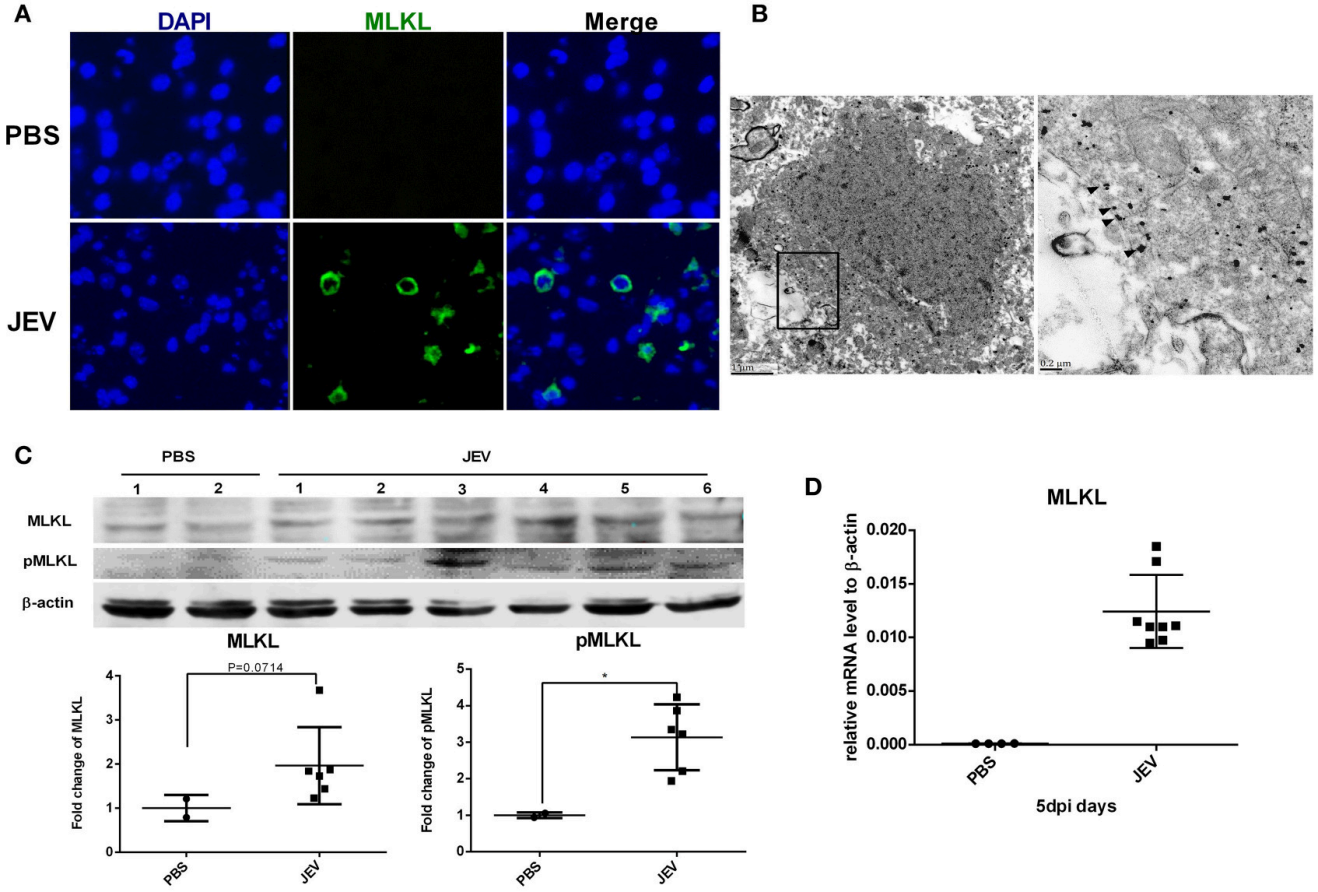
**MLKL mediated necroptosis is involved in JE**. Mice were infected i.p. with PBS or JEV 5 × 10^7^ PFU in 200 μl PBS/20 g. At 5 dpi, brains were harvested for immunochemistry, immunoelectromicroscopy, westeron blot and qRT-PCR. **(A)** The immunochemistry of MLKL in the brain sections of PBS or JEV administered mice (x400). There was obvious staining of MLKL around the plasma membrane in the brain sections of JEV infected mice while it was invisible in control mice. **(B)** Immuno-electron microscopic study of MLKL. The right panels show magnified regions of the boxed areas. Arrows showed the membrane localization of MLKL. **(C)** Western-blotting (upper) and quantitation (down) of protein MLKL and pMLKL in PBS or JEV administrated group. There was increased expression of protein MLKL and pMLKL in JEV infected group (PBS = 2, JEV = 6, ^*^P < 0.05). **(D)** The relative level of mRNA MLKL in PBS or JEV treated mice brain via qRT-PCR at 5 dpi. The data represents the relative mRNA level normalized with β-actin (PBS = 4, JEV = 8). The mRNA MLKL was significantly increased in JEV infected mouse brains compared with PBS group.

MLKL is the marker and executor of necroptosis. There was significantly increased expression of TNF-α after JEV infection *in vivo* and *in vitro* in our study (Supplementary Figure 2), which was in accordance with previous reports. Since TNF-α is the classical initiator of necroptosis through TNF-α/TNFR1/RIPK1/RIPK3/MLKL signaling (Cai et al., 2014; Wang et al., 2014), we detected the expression of MLKL in mice brain to identify whether MLKL mediated necroptosis was involved in JE. The MLKL around the plasma membrane was obvious in the brain sections of JEV infected mice while it was invisible in control mice (Figure 2A). Granules immunostained with anti-MLKL antibody were localized to the plasma membrane in the cortex of JEV infected mice according to the immune-electron microscopy (Figure 2B). Meanwhile, there was increased protein level of MLKL and pMLKL in JEV infected mice (Figure 2C). The level of MLKL mRNA was also increased in JEV infected group compared to the PBS treated mice (Figure 2D). Interestingly, the mice showing more serious clinical symptoms were detected to be higher in the level of MLKL mRNA (see Supplementary Figure 3). All these results demonstrated that MLKL mediated necroptosis was involved in JE.

### Necroptosis of neurons after JEV infection

**Figure 3:**
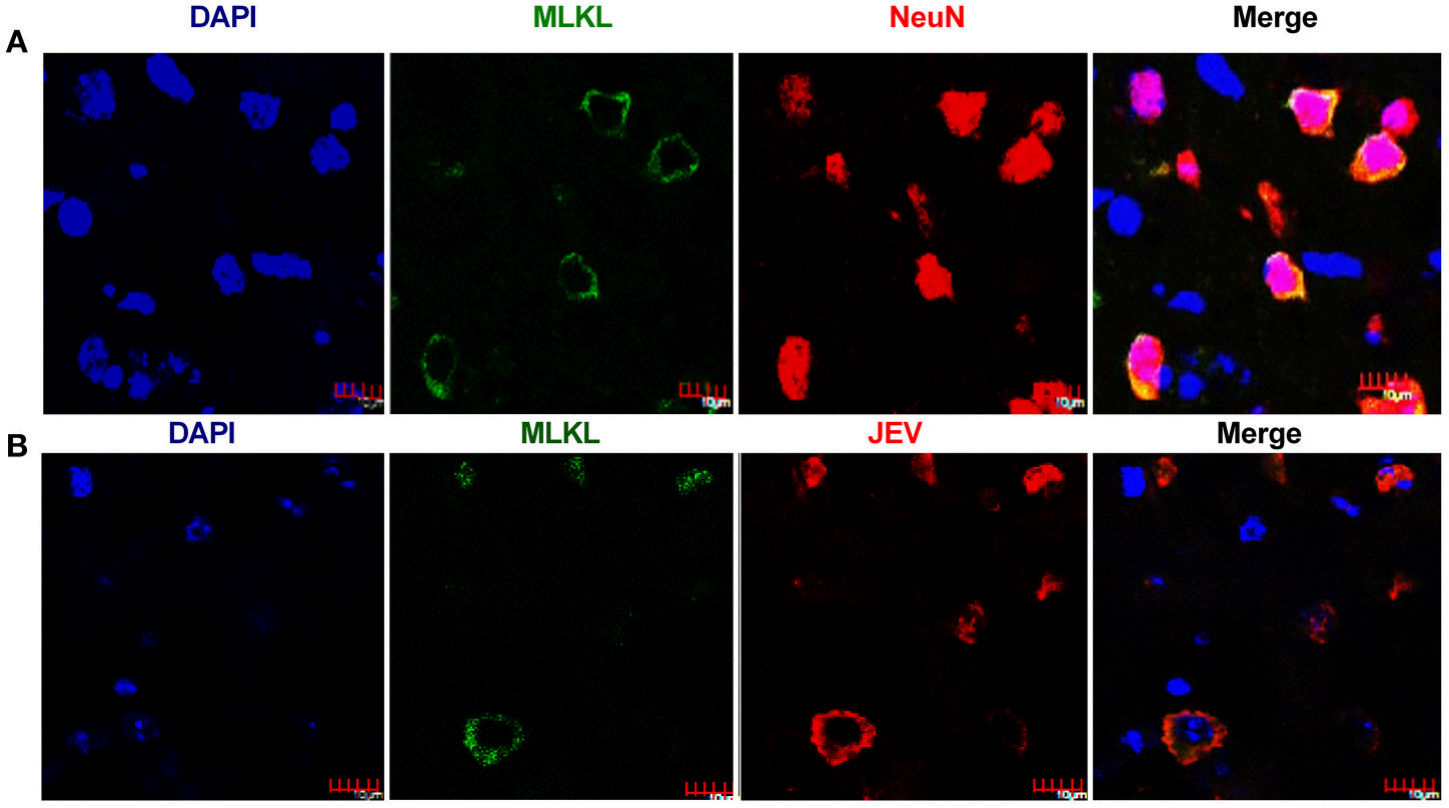
**Necroptosis of neurons after JEV infection**. The double-staining of MLKL and NeuN as well as the double-staining of JEV and MLKL was conducted in brain sections from JEV infected mice at 5 dpi. **(A)** Double-immunostaining of MLKL (green) and NeuN (red) in JEV infected mice. The results were acquired by confocal laser scanning microscope. The increased expression of MLKL was mainly occurred in neurons. **(B)** Double-immunostaining of MLKL (green) and JEV (red) in JEV infected mice. The expression of MLKL was closely correlated with JEV infection.

**Figure 4:**
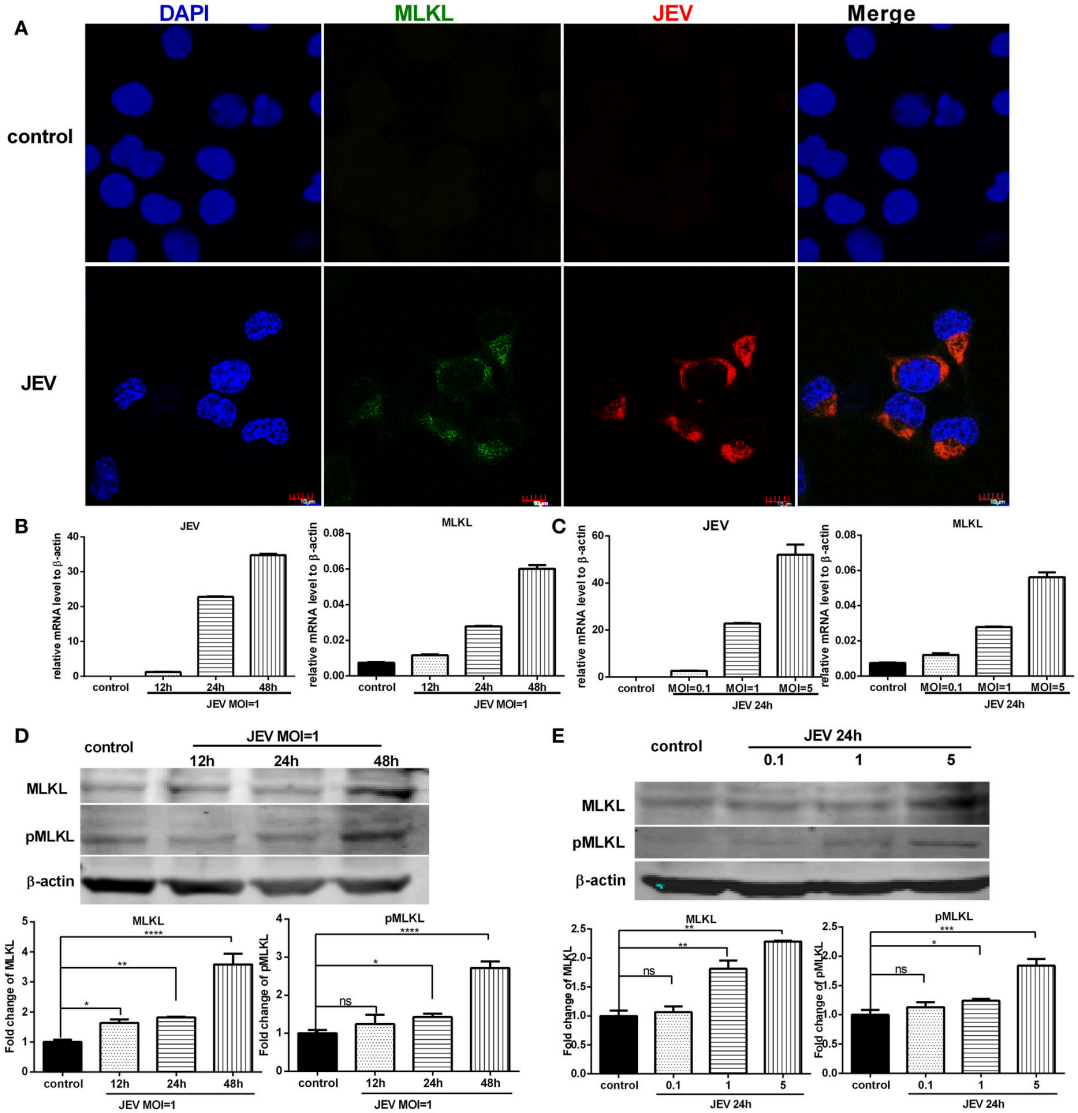
**The expression of MLKL is upregulated in neurons infected with JEV**. The expression of MLKL in Neuro2a cells was detected through immunofluorescence after JEV infection. Meanwhile, the mRNA level of MLKL and protein level of MLKL and pMLKL in Neuro2a cells were tested through qRT-PCR and western blotting after JEV infection at different MOI and infection time (The data represents the mean ± SEM for 3 independent experiments, ^*^P < 0.05, ^**^P < 0.01, ^***^P < 0.001 and ^****^P < 0.0001). **(A)** Double-immunostaining of MLKL (green) and JEV (red) in Neuro2a cells treated with DMEM (top) and infected with JEV MOI = 1, 24 h (bottom). **(B)** Neuro2a cells were infected with JEV at MOI = 1. And 12, 24, 48 h later, cells were collected for RNA extraction and qRT-PCR. The viral copies and the level of mRNA MLKL increased as the extension of infection time. **(C)** Neuro2a cells were infected with JEV at MOI = 0.1, 1, 5 respectively. And 24 h later, cells were collected for RNA extraction and qRT-PCR. The viral copies and the level of mRNA MLKL increased as the increase of infection dose. **(D)** Neuro2a cells were infected with JEV at MOI = 1. And 12, 24, 48 h later, cells were collected for total protein extraction and western-bloting. With the extension of infection time, the expression of protein MLKL and pMLKL increased. **(E)** Neuro2a cells were infected with JEV at MOI = 0.1, 1, 5 respectively. And 24 h later, cells were collected for total protein extraction and western-bloting. The level of protein MLKL and pMLKL increased as the increase of infection dose.

Neuronal death played crucial roles in the pathogenesis of JE. To test whether necroptosis occurs in the neurons, the double-staining with MLKL and NeuN (the marker of neurons) was carried out in mouse brains. As expect, the upregulation of MLKL were mostly occurred in NeuN-labeling cells (Figure 3A). This indicated that MLKL mediated necroptosis mainly occurred in neurons during JEV infection. Meanwhile, there were also co-localization of JEV and MLKL in the cytoplasm of the neurons (Figure 3B) which was also observed in the cell line Neuro2a cells (Figure 4A).

### The expression of MLKL is upregulated in neurons infected with JEV

It has been shown that MLKL is a mediator of necroptosis. To investigate whether the upregulation of MLKL expression was caused by JEV infection, the immunostaining of MLKL and JEV in Neuro2a cells was conducted. *In vitro*, there was visible increased expression of MLKL in Neuro2a cells infected with JEV compared with control (Figure 4A). Furtherly, the expression of MLKL in mRNA (Figures 4B,C) and protein level (Figures 4D,E) were upregulated accompanying the increase of JEV infection time and dose. In summary, JEV infection promoted the expression of MLKL.

### MLKL deleted mice show alleviated progression of JE

**Figure 5:**
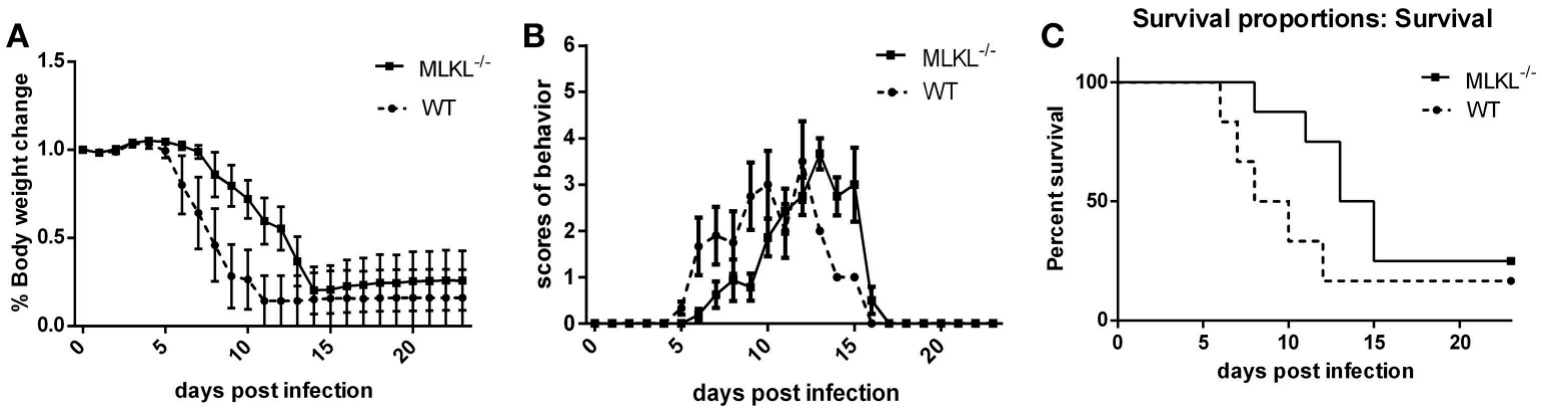
**MLKL deleted mice show alleviated progression of JE**. Wild and MLKL^−/−^ mice were administered with JEV 5 × 10^7^ PFU/20 g in 200 μl PBS intraperitoneally. The body weight, behavior scores and survival rate were recorded daily from 0 dpi to 23 dpi when the survivals were completely stable (WT = 12, MLKL^−/−^ = 16). **(A)** The weight of each mouse was measured and recorded at 16: 30–17: 00 (the data represents the mean ± SD). Compared with wild mice, MLKL^−/−^ group showed alleviated weight loss. **(B)** The behavior score was recorded twice every day according to the scoring criteria (0 = no piloerection, no restriction of movement, no body stiffening, no hind limb paralysis. 1 = piloerection, no restriction of movement, no body stiffening, no hind limb paralysis. 2 = piloerection, restriction of movement, no body stiffening, no hind limb paralysis. 3 = piloerection, restriction of movement, body stiffening, no hind limb paralysis. 4 = piloerection, restriction of movement, body stiffening, hind limb paralysis. 5 = piloerection, restriction of movement, body stiffening, hind limb paralysis, sometimes tremor even death. And the dead mice were excluded from the cohort and the mean scores of all mice in each group were calculated. The data represent the mean ± SD). MLKL^−/−^ mice showed slowed onset and progression of JE compared with wild group. **(C)** The death and survival of mice in each group was recorded and the data was analyzed and shown as Kaplan–Meier survival curves. Even though there was no significant difference in the final survival rate, MLKL^−/−^ group survived for longer time than wild group.

To explore the role of MLKL in the progression of JE *in vivo*. MLKL knock-out mice were infected with JEV, the body weight (Figure 5A), behavior scores (Figure 5B), and survival rate (Figure 5C) were recorded daily from 0 dpi until all the survivors were completely stable. Although, there was no significant improvement in final survival rate in MLKL^−/−^ mice compared with wild mice, MLKL^−/−^ mice showed delayed onset of JE and alleviated symptoms. Thus, MLKL mediated necroptosis aggravated the progression of JE.

### MLKL deleted mice show decreased inflammatory cytokines during JEV infection

**Figure 6:**
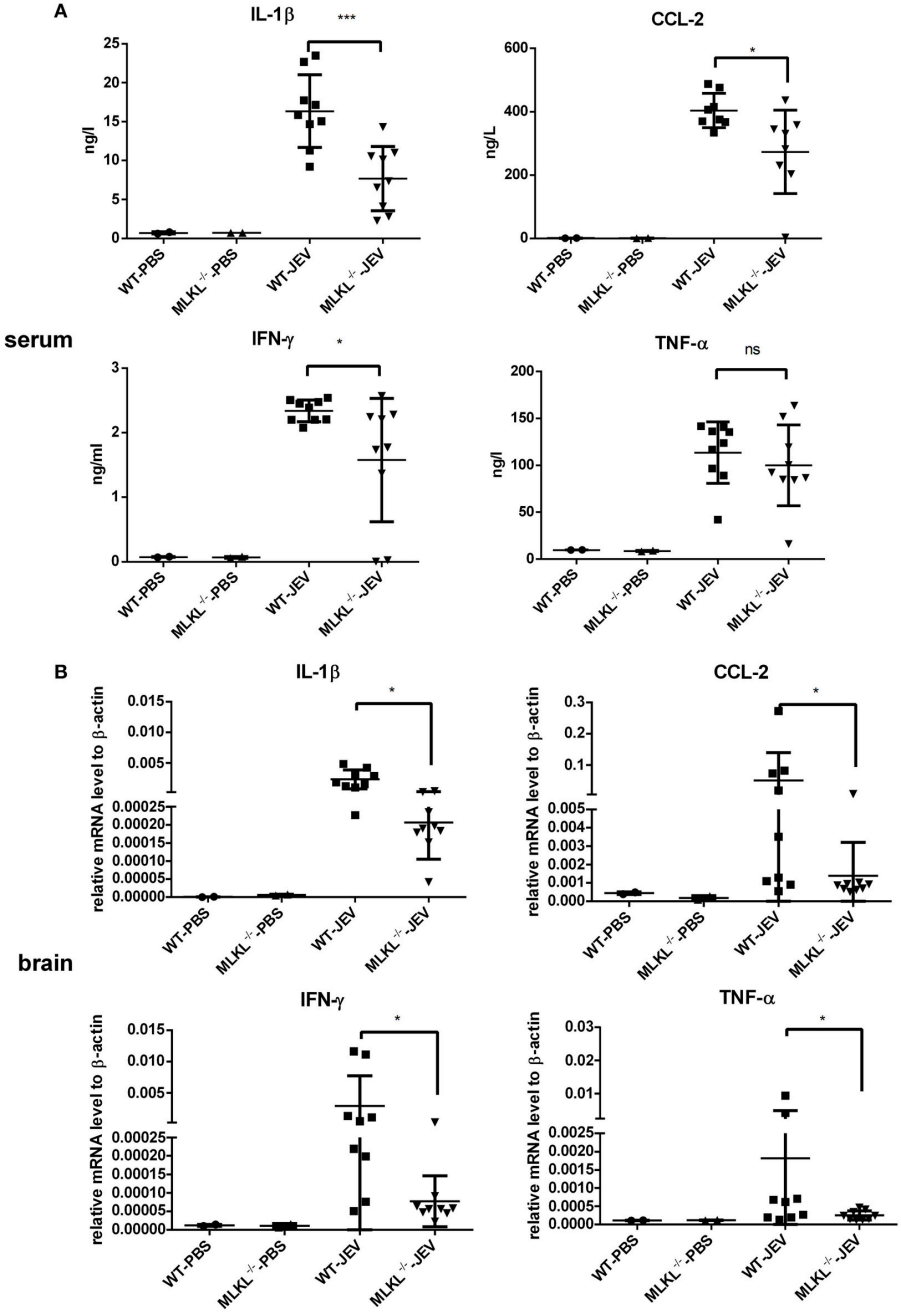
**MLKL deleted mice show decreased inflammatory cytokines during JEV infection**. The serum and brain from each mice in MLKL^−/−^ and wild group were collected at 5 dpi, and the main inflammatory cytokines in serum and brain were tested. **(A)** The level of CCL-2, IL-1β, IFN-γ, TNF-α in the serum of WT or MLKL^−/−^ mice was detected by ELISA assay kit (WT-PBS = 2, MLKL^−/−^-PBS = 2, WT-JEV = 9, MLKL^−/−^-PBS = 9). **(B)** The expression of CCL-2, IL-1β, IFN-γ, TNF-α in the brain of WT or MLKL^−/−^ mice was detected by qRT-PCR (WT-PBS = 2, MLKL^−/−^-PBS = 2, WT-JEV = 9, MLKL^−/−^-PBS = 9, ^*^P < 0.05, ^***^P < 0.001).

In the MLKL mediated necroptosis, massive DAMPs released from the disintegrated cells trigger and accelerate inflammation. To explore the change of inflammation between wild and MLKL^−/−^ mice after JEV infection, the level of some inflammatory cytokines in the serum (Figure 6A) and brain (Figure 6B) at 5 dpi were tested. There was decreased level of IL-1β, CCL-2, IFN-γ in the serum of MLKL^−/−^ mice compared with wild mice. The level of TNF-α also showed decline to some extent, even though without statistically significant difference. The expression of CCL-2, IL-1β, IFN-γ, and TNF-α were decreased in the brains of MLKL^−/−^ mice than wild mice. The decreased inflammation might contribute to the alleviated progression of JE. At the same time, the death of neuron was also diminished in MLKL^−/−^ mice at 5 dpi (see Supplementary Figure 4). However, there was no significant difference of the viral load in the brain between MLKL^−/−^ and wild mice (see Supplementary Figure 5). Thus, MLKL mediated necroptosis worsened the inflammation during the JEV infection.

## Discussion

Neuronal death plays fatal roles in the progression of neuroinflammation during JEV infection, among which apoptosis and autophagy have been demonstrated to be involved in. The neurons can directly undergo apoptosis due to viral replication or through a bystander mechanism where overactivation of microglia and astrocytes leads to the release of numerous proinflammatory cytokines, including TNF-α, IL-1β, and IL-6 (Swarup et al., 2007). The existence of massive neuronal necrosis in the human fatal cases and animal models has also been reported. Recently, it was found that necroptosis also participated in many CNS diseases (Dhib-Jalbut and Kalvakolanu, 2015; Ito et al., 2016; Meessen-Pinard et al., 2017). It can be triggered by the TNF family of cytokines and by ligands of Toll-like receptors 3 and 4 (Vandenabeele et al., 2010). However, it is still unclear whether the necroptosis, as a particular form of necrosis, participates in the pathology of JE. In this study, we demonstrated for the first time that MLKL mediated necroptosis was involved in the pathogenesis of JE. Meanwhile, the expression of MLKL was increased during JEV infection. MLKL^−/−^ mice showed delayed onset of JE, alleviated symptoms and decreased neuroinflammation and proinflammatory cytokines compared with wild mice after JEV infection.

Currently, the research of necroptosis pathway is mainly focused on TNF/TNFR/RIPK1/RIPK3/MLKL and RIPK1 independently DAI/RIPK3/MLKL signaling. As reported, production of TNF-α and stimulation of TLRs participated in the initiation of MLKL mediated necroptosis (Vandenabeele et al., 2010). During JEV infection, there is robust production of TNF-α, which might trigger the necroptosis. Many approaches have been developed to assess the contribution of necroptosis in experimental disease models such as detection of micro-morphological and biochemical features, measurement of MLKL expression, assessment of phosphorylated MLKL level and so on, among which MLKL phosphorylation has been used as a diagnostic biomarker (Jouan-Lanhouet et al., 2014). In this study, we detected MLKL around the neuron membrane from JEV-infected mice and the increased expression of MLKL and pMLKL in JEV infected mice and cells. Meanwhile, the mRNA level of MLKL was also increased after JEV infection. In general, we proved the existence of MLKL mediated necroptosis during JE. Interestingly, we also found that the increased expression of MLKL was mainly occurred in JEV infected cells *in vitro* and *in vivo*. We speculate that JEV infection might induce the expression of MLKL and several host factors might promote the phosphorylation of MLKL at the same time. However, the exact trigger and signal of MLKL phosphorylation during JEV infection is still under investigation.

It has been established that necroptosis restricts propagation and spread of lytic virus such as HSV-1 and IAV by eliminating the infected cells as well as exaggerating inflammatory response (Huang et al., 2015; Kuriakose et al., 2016; Nogusa et al., 2016). While, as reported, there was no significantly difference in the viral load between WT and necroptosis blocking mice infected with IAV until 6 dpi (Kuriakose et al., 2016). In our study, the viral load between wild and MLKL^−/−^ mice after direct injection 50 PFU JEV into the brains showed no significant difference (see Supplementary Figure 6) before they gradually died. Thus, necroptosis alone was not enough to restrict JEV spread *in vivo*. Conversely, necroptosis and inflammation promoted each other and caused more serious CNS disorders and death during JE.

JE is characterized with extensive neuroinflammation. Until now, there is no special or effective therapy but symptomatic relief and supportive treatment (Misra and Kalita, 2010). The DAMPs, activated glia, inflammatory cytokines and breakdown of BBB forming an uncontrolled damaged microenvironment lead to excessive neuronal death, brain dysfunctions, cerebral edema and respiratory failure, which is the main cause of death during JE (Han et al., 2014). Blocking the vicious cycle of inflammatory damage has been shown to benefit the therapy of JE (Ye et al., 2014; Li et al., 2015). Recently, necroptosis has been demonstrated to lead to disruption of cell membranes and release of cytoplasmic contents which triggers and exacerbates inflammation response (Silke et al., 2015). Several studies have shown that suppression of necroptosis can alleviate inflammation induced injury. Kuriakose et al. (2016) reported that blocking necroptosis could reduce inflammatory responses and epithelial damage and protect mice from mortality during IAV infection. Consistently, in our study, MLKL^−/−^ mice showed delayed onset of JE and alleviated symptoms compared with wild mice after JEV infection. It indicated that MLKL mediated necroptosis aggravated the progression of JE. Although MLKL^−/−^ mice did not survive from JE totally, we found that MLKL^−/−^ mice showed decreased level of CCL-2, IL-1β, IFN-γ in brain and serum compared with wild mice after JEV infection. Thus, the slowing of JE progression in MLKL^−/−^ mice could be attributed to the interruption of the vicious cycle of necroptosis-induced inflammation.

In summary, our study indicated that MLKL mediated necroptosis was involved in the pathogenesis of JE. Targeting necroptosis may provide new ways to the development of novel therapeutic strategies for the treatment of JEV induced encephalitis.

## Author contributions

PB: Conception and design, data collection and assembly, data analysis and interpretation, manuscript writing. XZ, LW: Data analysis and interpretation, manuscript writing. CY: Data collection and assembly. HF, YC: Data collection. YZ, FZ: Administrative support, provision of study material. ZJ: Conception and design, financial support, administrative support, final approval of manuscript. YL: Conception and design, financial support, administrative support, data analysis and interpretation, manuscript writing. All authors read and approved the final manuscript.

## Funding

This work was supported by the National Natural Science Foundation of China (NSFC) (Grant no. 31370193) and the National Key State Science and Technology Projects of China (Grant no. 2013ZX10004609).

### Conflict of interest statement

The authors declare that the research was conducted in the absence of any commercial or financial relationships that could be construed as a potential conflict of interest.

## Supplementary material

Supplementary Figure 1 **Massive antigen of JEV was detected in JEV infected mouse brains at 5 dpi**.

Supplementary Figure 2 **The relative expression of mRNA TNF-α after JEV infection *in vivo* and *in vitro***. The brains of mice were harvested at 2, 4, 6 dpi after JEV infection intraperitoneally and the RNA of the whole brain was extracted and the level of TNF-α was detected through qRT-PCR (PBS = 2, 2 dpi = 2, 4 dpi = 2, 6 dpi = 4). And Neuro2a cells were collected after JEV infection at different MOI (0.1, 1, and 5) and infection time (12, 24, and 48 h) for the detection of TNF-α (The data represent the mean ± SEM for 3 independent experiments). **(A)** The expression of TNF-α in mouse brains at 2, 4, 6 dpi after JEV infection. **(B)** The expression of TNF-α in Neuro2a cells at 12, 24, 48 h after JEV infection at MOI = 1. **(C)** The expression of TNF-α in Neuro2a cells at MOI = 0.1, 1, 5 at 24 h after JEV infection.

Supplementary Figure 3 **The level of mRNA MLKL was higher in mice with severe clinical symptoms in wild mice**.

Supplementary Figure 4 **At 5 dpi, mice from WT-PBS, MLKL^−/−^-PBS, WT-JEV, MLKL^−/−^-JEV were sacrificed for PI staining. (A)** The representative images of PI staining of brain sections from each group (100x). **(B)** The intensity of PI positively stained cells of each group were analyzed with Image J (Data represents mean ± SEM. WT-PBS = 2, MLKL^−/−^-PBS = 2, WT-JEV = 3, MLKL^−/−^-JEV = 3, 3 sections per mouse, 5 fields per section).

Supplementary Figure 5 **The relative viral load in the brain of WT or MLKL^−/−^ mice were detected by qRT-PCR at 5 dpi after administration with JEV 5 × 107 PFU/20g in 200 μl PBS intraperitoneally (WT-JEV = 9, MLKL^−/−^-JEV = 9)**.

Supplementary Figure 6 **The relative viral load in the brains of WT or MLKL^−/−^ mice after injection 50 PFU JEV into the brains**. Mice were anesthetized with 2% pentobarbital sodium (0.05 ml/10 g body weight) and transferred to a stereotaxic apparatus (Stoelting Co, Wood Dale, IL, USA). Then 50 PFU of virus in 2 μl PBS was injected into the right cerebral cortex about 2 mm below the dura through a 5-μL microsyringe (Gaoge China). At 2, 4, 6 dpi, brains of each group were harvested and the relative viral load in the brain was detected by qRT-PCR (2 dpi WT-JEV = 4, MLKL^−/−^-JEV = 4; 4 dpi WT-JEV = 4, MLKL^−/−^-JEV = 4; 6 dpi WT-JEV = 6, MLKL^−/−^-JEV = 7).

Supplementary Table 1 **The antibodies used in this study**. For IHC, the primary antibodies were diluted with 0.01 M PBS containing 0.1% Triton X-100 and 1% bovine serum albumin (BSA). For WB, the primary antibodies were diluted with 0.01 M PBS containing 3% BSA.
