# Supplementary figures and images for: MLKL Mediated Necroptosis Accelerates JEV-Induced Neuroinflammation in Mice
Source: Front Microbiol. 2017 Feb 28;8:303. doi: 10.3389/fmicb.2017.00303 (PMC5328978; doi:10.3389/fmicb.2017.00303)

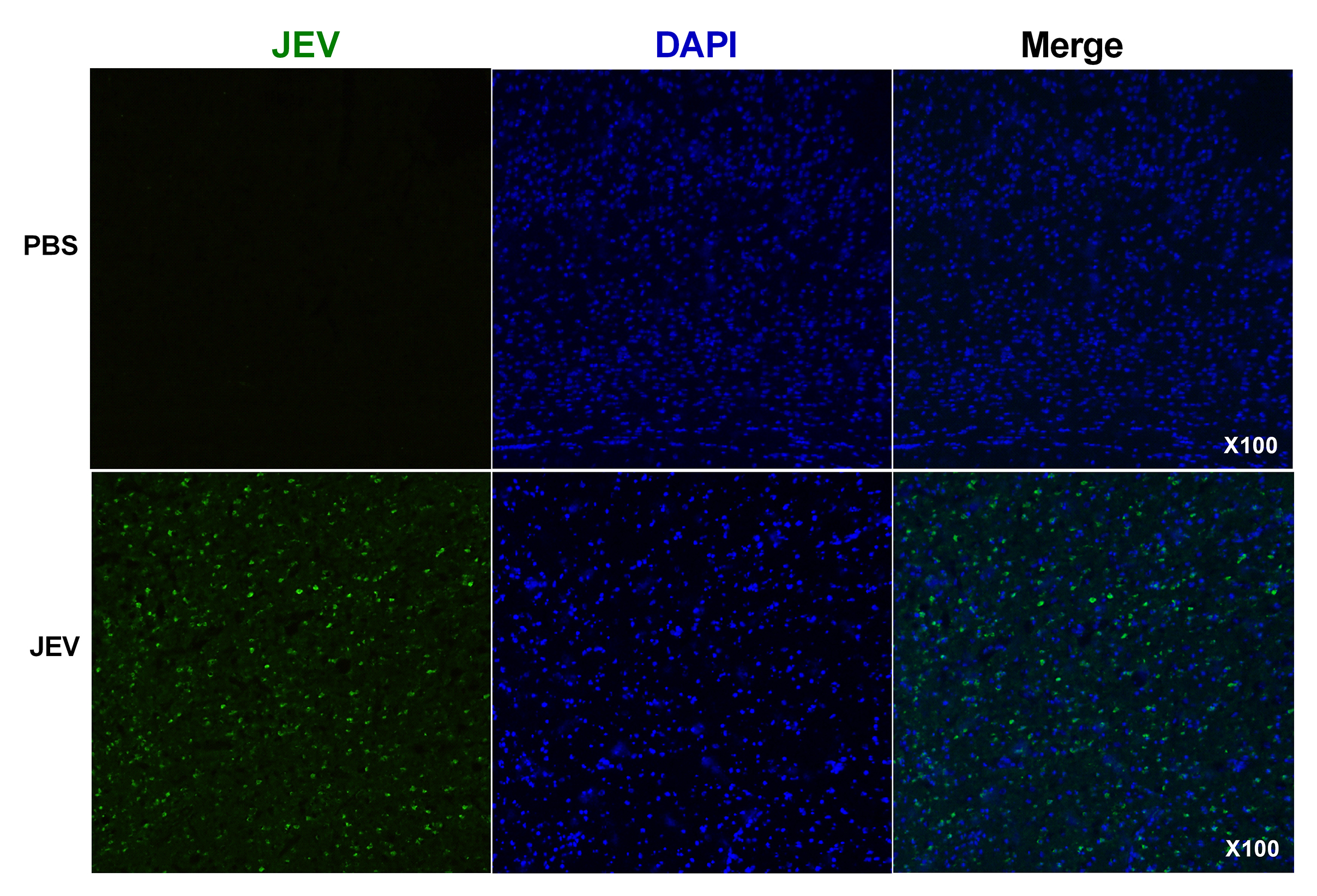

Supplement: Supplementary Figure 1 — Massive antigen of JEV was detected in JEV infected mouse brains at 5 dpi. [file Image1.JPEG]

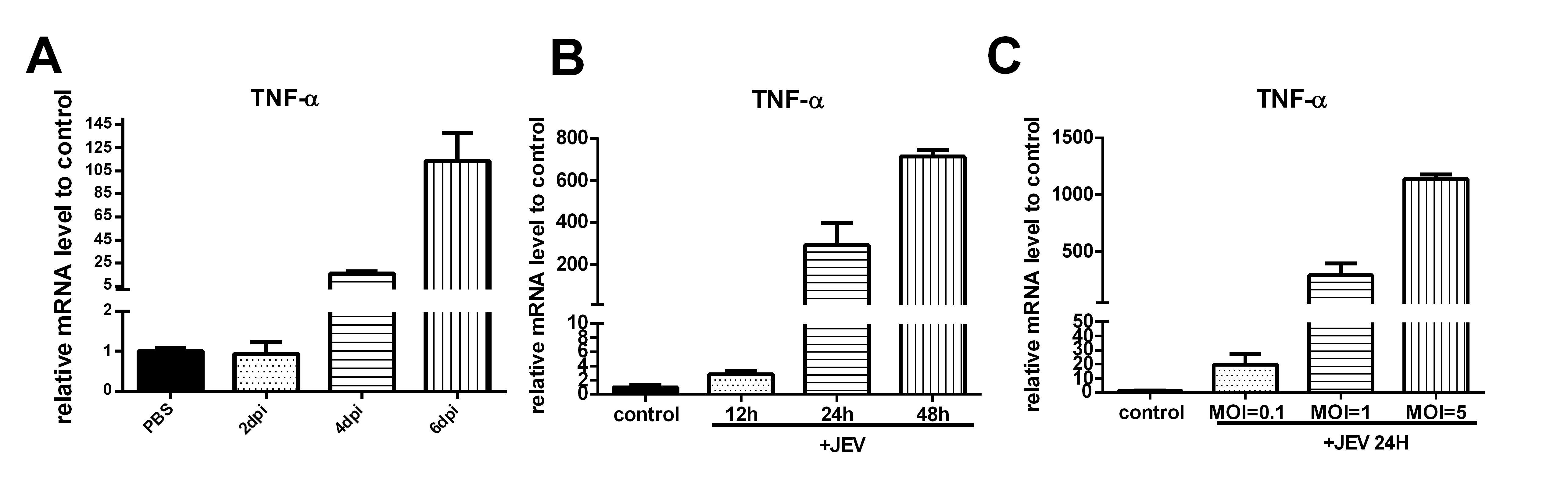

Supplement: Supplementary Figure 2 — The relative expression of mRNA TNF-α after JEV infection in vivo and in vitro. The brains of mice were harvested at 2, 4, 6 dpi after JEV infection intraperitoneally and the RNA of the whole brain was extracted and the level of TNF-α was detected through qRT-PCR (PBS = 2, 2 dpi = 2, 4 dpi = 2, 6 dpi = 4). And Neuro2a cells were collected after JEV infection at different MOI (0.1, 1, and 5) and infection time (12, 24, and 48 h) for the detection of TNF-α (The data represent the mean ± SEM for 3 independent experiments). (A) The expression of TNF-α in mouse brains at 2, 4, 6 dpi after JEV infection. (B) The expression of TNF-α in Neuro2a cells at 12, 24, 48 h after JEV infection at MOI = 1. (C) The expression of TNF-α in Neuro2a cells at MOI = 0.1, 1, 5 at 24 h after JEV infection. [file Image2.JPEG]

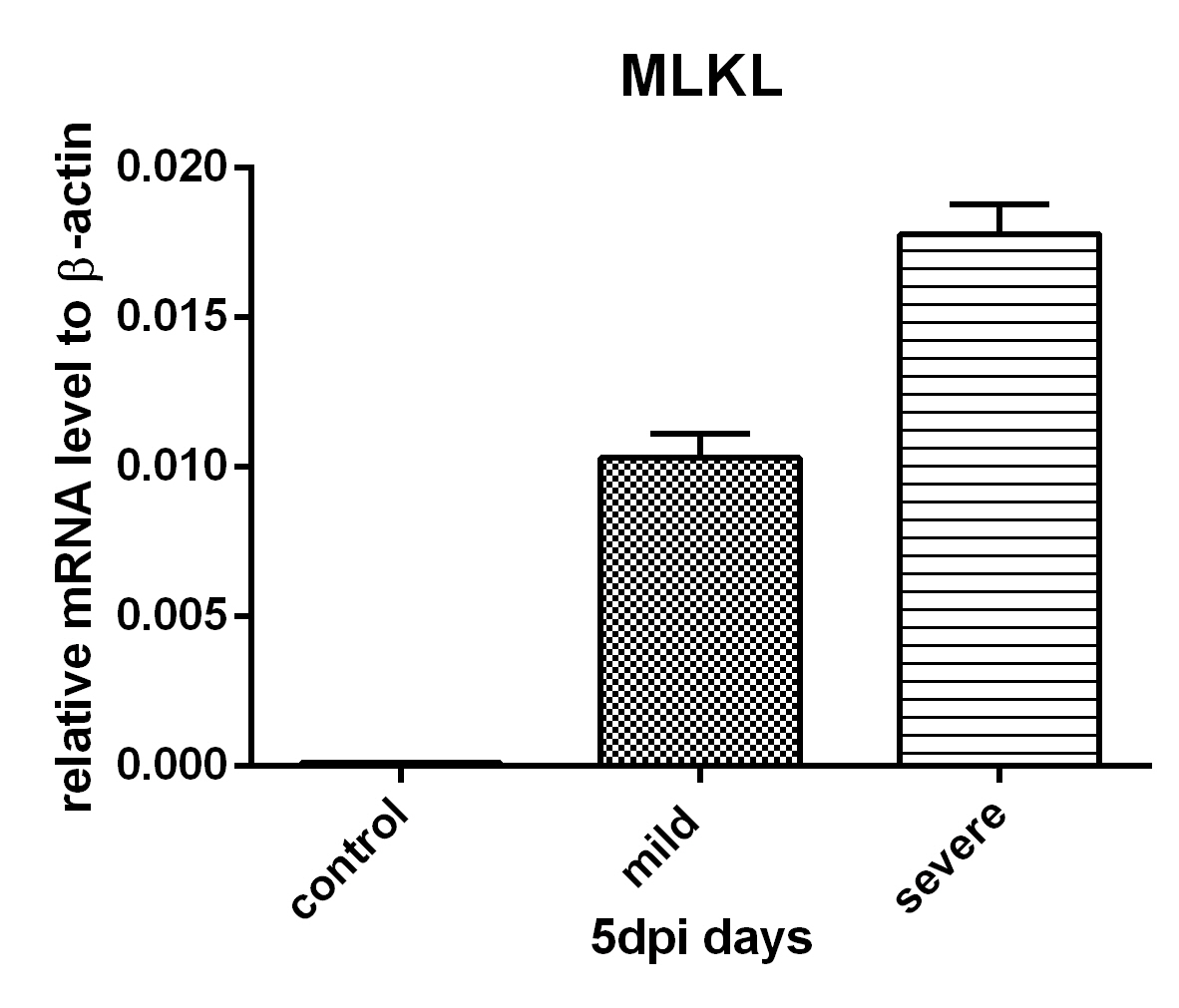

Supplement: Supplementary Figure 3 — The level of mRNA MLKL was higher in mice with severe clinical symptoms in wild mice. [file Image3.JPEG]

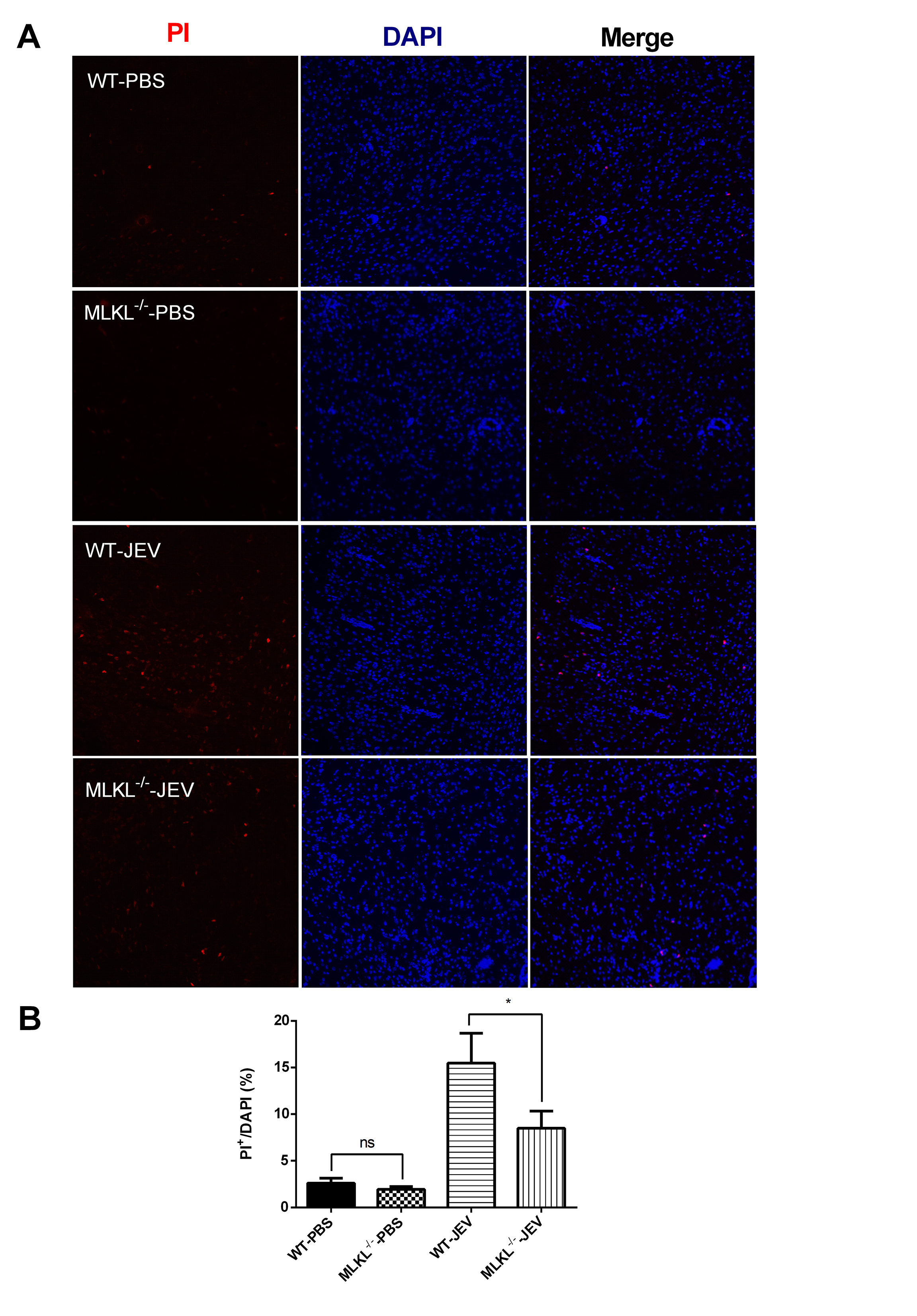

Supplement: Supplementary Figure 4 — At 5 dpi, mice from WT-PBS, MLKL−/−-PBS, WT-JEV, MLKL−/−-JEV were sacrificed for PI staining. (A) The representative images of PI staining of brain sections from each group (100x). (B) The intensity of PI positively stained cells of each group were analyzed with Image J (Data represents mean ± SEM. WT-PBS = 2, MLKL−/−-PBS = 2, WT-JEV = 3, MLKL−/−-JEV = 3, 3 sections per mouse, 5 fields per section). [file Image4.JPEG]

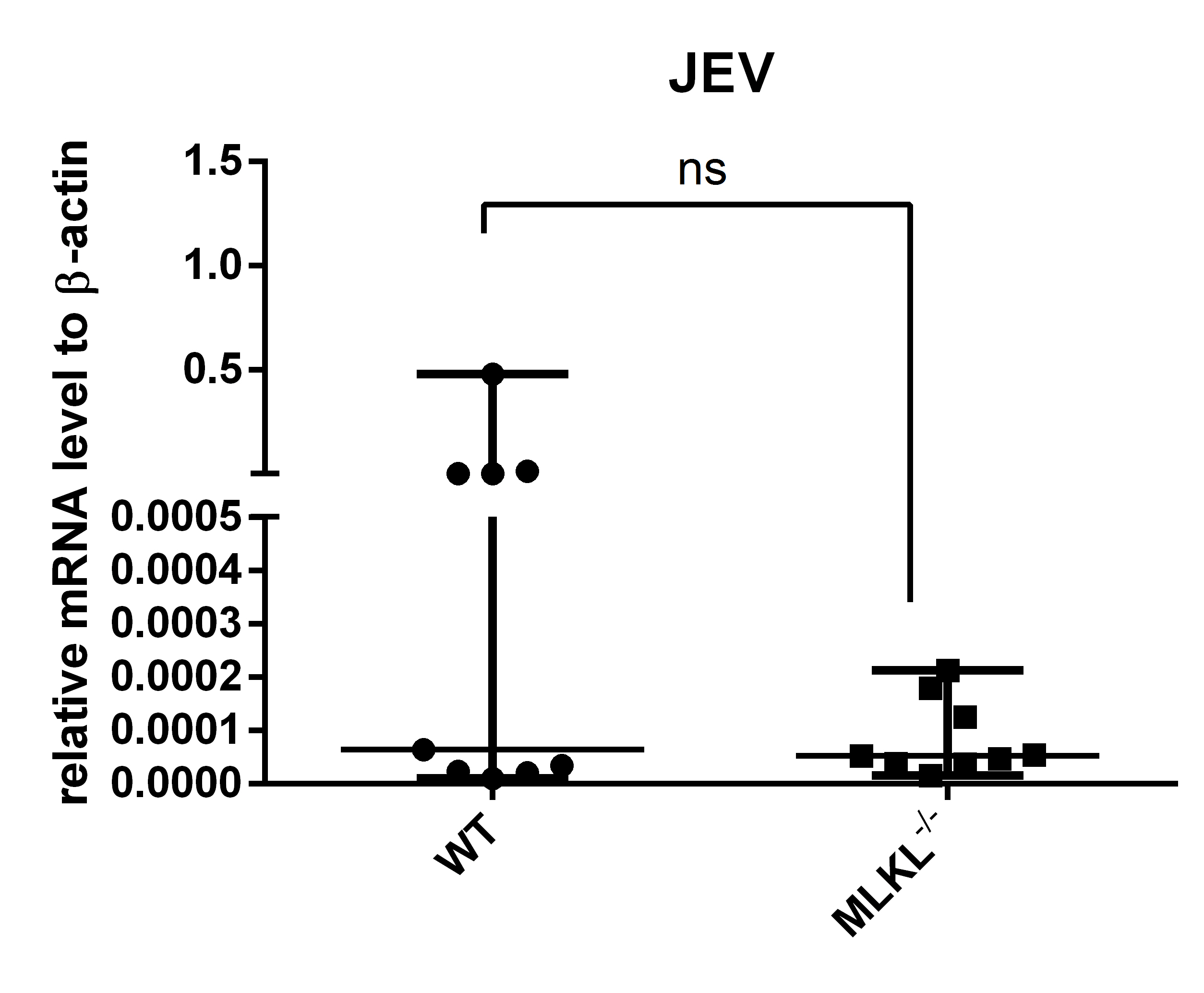

Supplement: Supplementary Figure 5 — The relative viral load in the brain of WT or MLKL−/− mice were detected by qRT-PCR at 5 dpi after administration with JEV 5 × 10^7^ PFU/20g in 200 μl PBS intraperitoneally (WT-JEV = 9, MLKL−/−-JEV = 9). [file Image5.JPEG]

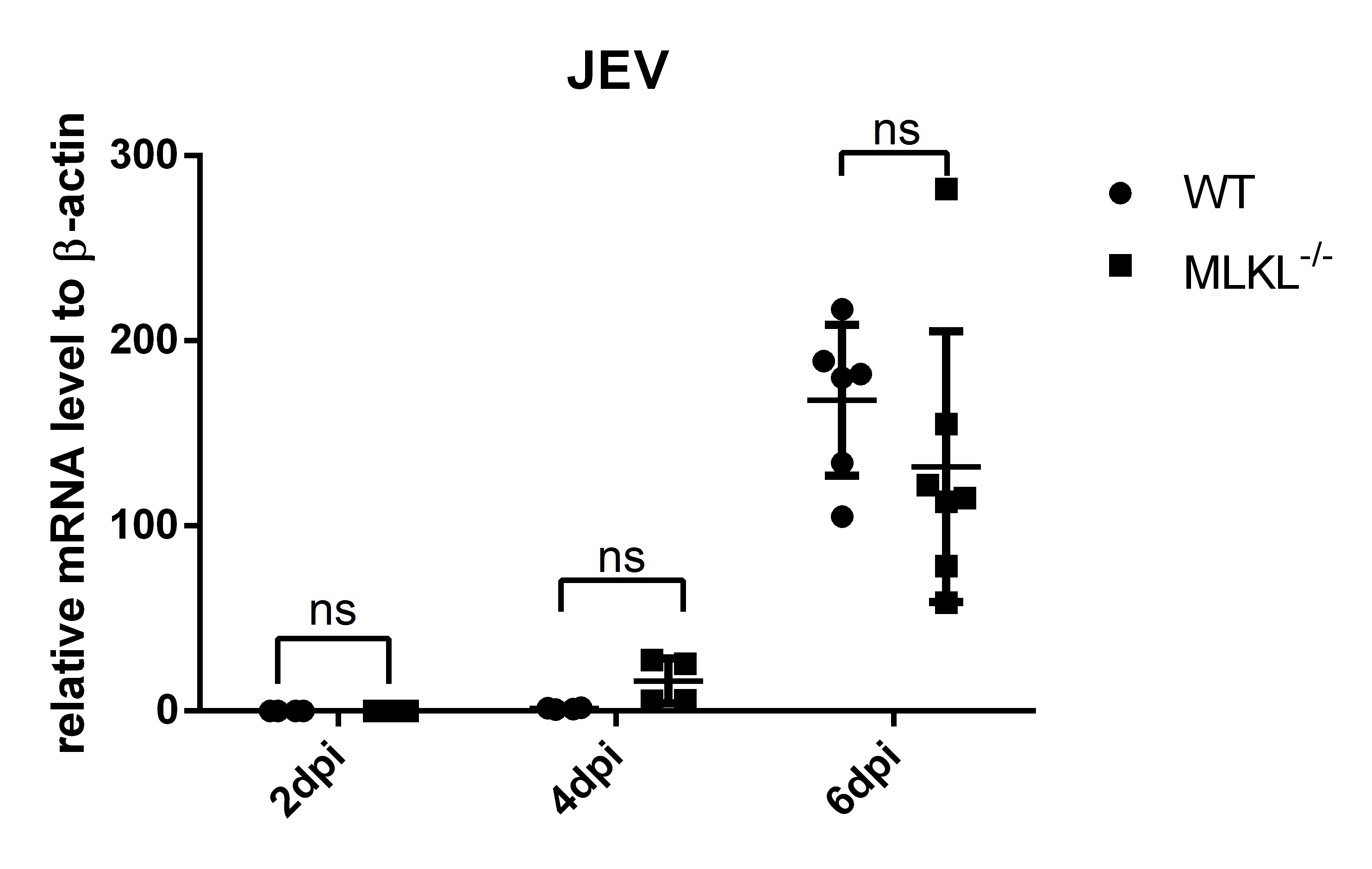

Supplement: Supplementary Figure 6 — The relative viral load in the brains of WT or MLKL−/− mice after injection 50 PFU JEV into the brains. Mice were anesthetized with 2% pentobarbital sodium (0.05 ml/10 g body weight) and transferred to a stereotaxic apparatus (Stoelting Co, Wood Dale, IL, USA). Then 50 PFU of virus in 2 μl PBS was injected into the right cerebral cortex about 2 mm below the dura through a 5-μL microsyringe (Gaoge China). At 2, 4, 6 dpi, brains of each group were harvested and the relative viral load in the brain was detected by qRT-PCR (2 dpi WT-JEV = 4, MLKL−/−-JEV = 4; 4 dpi WT-JEV = 4, MLKL−/−-JEV = 4; 6 dpi WT-JEV = 6, MLKL−/−-JEV = 7). [file Image6.JPEG]
