# Supplementary material for: MLKL Mediated Necroptosis Accelerates JEV-Induced Neuroinflammation in Mice
Source: Front Microbiol. 2017 Feb 28;8:303. doi: 10.3389/fmicb.2017.00303 (PMC5328978; doi:10.3389/fmicb.2017.00303)
Supplement: Supplementary Table 1 — The antibodies used in this study. For IHC, the primary antibodies were diluted with 0.01 M PBS containing 0.1% Triton X-100 and 1% bovine serum albumin (BSA). For WB, the primary antibodies were diluted with 0.01 M PBS containing 3% BSA. [file Table1.DOCX]

| **Antibodies** | **Hosts** | **Dilutions** | **Sources** |
| --- | --- | --- | --- |
| Anti-MLKL | Rat | IHC 1:800  WB 1:500 | Millipore, Billerica, MA, USA |
| Anti-NeuN | Rabbit | IHC 1:900 | Abcam England |
| Anti-JEV | Mouse | IHC 1:100 | China |
| Anti-β-actin | Mouse | WB 1:1000 | Proteintech China |
| Anti-pMLKL | Rabbit | WB 1:800 | Abcam England |
| FITC-anti-mouse IgG | Rabbit | IHC 1:200 | Proteintech China |
| Cy3-anti-rabbit IgG | Donkey | IHC 1:200 | Proteintech China |
| Cy3-anti-mouse IgG | Goat | IHC 1:200 | Proteintech China |
| FITC-anti-rat IgG | Donkey | IHC 1:200 | Proteintech China |
| DyLight 680-anti-rabbit IgG | Goat | WB 1:10000 | BD USA New Jersey |
| DyLight 680-anti-rat IgG | Goat | WB 1:10000 | BD USA New Jersey |
| DyLight 800-anti-mouse IgG | Goat | WB 1:10000 | BD USA New Jersey |

Supplement table 1. The antibodies used in this study.

For IHC, the primary antibodies were diluted with 0.01 M PBS containing 0.1 % Triton X-100 and 1 % bovine serum albumin (BSA). For WB, the primary antibodies were diluted with 0.01 M PBS containing 3 % BSA.
